# Visual Cancer Communication on Social Media: An Examination of Content and Effects of #Melanomasucks

**DOI:** 10.2196/10501

**Published:** 2018-09-05

**Authors:** Hyunyi Cho, Nathan Silver, Kilhoe Na, Dinah Adams, Kate T Luong, Chi Song

**Affiliations:** ^1^ School of Communication The Ohio State University Columbus, OH United States; ^2^ Futurety LLC Columbus, OH United States; ^3^ Division of Biostatistics The Ohio State University Columbus, OH United States

**Keywords:** cancer, comments, common sense model, emotions, illness perception, Instagram, likes, melanoma, participative engagement, social media, social sharing, social support, visual communication

## Abstract

**Background:**

Instagram is increasingly becoming a platform on which visual communication of cancer takes place, but few studies have investigated the content and effects. In particular, a paucity of research has evaluated the effects of visual communication of cancer on participative engagement outcomes.

**Objective:**

The objective of our study was to investigate cancer-related beliefs and emotions shared on Instagram and to examine their effects on participative engagement outcomes including likes, comments, and social support.

**Methods:**

This study analyzed the content of 441 posts of #melanomasucks on Instagram and assessed the effects of the content characteristics on outcomes, including the number of likes and comments and types of social support using group least absolute shrinkage and selection operator logistic regression.

**Results:**

Posts about controlling melanoma were most frequent (271/441, 61.5%), followed by 240 (54.4%) posts about outcomes of having melanoma. Ninety posts (20.4%) were about the causes of melanoma. A greater number of posts expressed positive (159/441, 36.1%) than negative emotions (100/441, 22.7%). Eighty posts (18.1%) expressed hope, making it the most frequently expressed emotion; 49 posts expressed fear (11.1%), 46 were humorous (10.4%), and 46 showed sadness (10.4%). Posts about self behavior as a cause of melanoma decreased likes (*P*<.001) and social support comments (*P*=.048). Posts about physical consequences of melanoma decreased likes (*P*=.02) but increased comments (*P*<.001) and emotional social support (*P*<.001); posts about melanoma treatment experience increased comments (*P*=.03) and emotional social support (*P*<.001). None of the expressions of positive emotions increased likes, comments, or social support. Expression of anger increased the number of likes (*P*<.001) but those about fear (*P*<.001) and joy (*P*=.006) decreased the number of likes. Posts about fear (*P*=.003) and sadness (*P*=.003) increased emotional social support. Posts showing images of melanoma or its treatment on the face or body parts made up 21.8% (96/441) of total posts. Inclusion of images increased the number of comments (*P*=.001).

**Conclusions:**

To our knowledge, this is the first investigation of the content and effects of user-generated visual cancer communication on social media. The findings show where the self-expressive and social engagement functions of #melanomasucks converge and diverge, providing implications for extending research on the commonsense model of illness and for developing conceptual frameworks explaining participative engagement on social media.

## Introduction

### Background

Instagram is rapidly becoming a public platform where images, beliefs, and emotions about cancer are expressed and shared. At the time of this writing, there are millions of Instagram posts about various cancer experiences. One of the fastest growing platforms of social media, following only Facebook [1], Instagram is unique in that visual images occupy a significant portion of a post’s space. Instagram is also more public compared with Facebook. Whereas on Facebook sharing is based on friendships, on Instagram it is not. Visual images such as those posed on Instagram have the potential to significantly impact cancer communication processes through increased exposure, attention, emotional engagement, and memorability [2,3]. Self-expressions of cancer experiences on Instagram can be viewed worldwide, fostering far reaching impacts on social perceptions and discourse about cancer. In particular, these efforts are assisted by hashtags (#) for message labeling, movement organizing, and visibility, searchability, and documentability of grassroots content [4].

Despite this popularity, visual communication of cancer on social media remains underinvestigated. Extant research on social media content related to cancer has focused on text-based content, such as that on Twitter [5,6]. Only a limited number of studies have investigated visual cancer communication on social media [7,8], and few have examined the effects of visual cancer communication on social media. Notably, a 2018 study investigated the effects of vaccine images on retweets [9]. Aiming to fill this void in knowledge, this study investigated the illness beliefs and emotions expressed under #melanomasucks on Instagram and their effects on participative engagement outcomes, including likes, comments, and social support.

### Commonsense Representations of Illness Beliefs and Emotions

Melanoma is the fifth most prevalent cause of cancer death affecting both men and women in the United States [10]. Researchers view that the increase in melanoma incidence may be associated with a range of factors, including behavioral and environmental factors [11,12]. Melanoma is particularly relevant to this investigation of visual cancer communication because it begins on the surface of body, whereas most other cancers involve internal organs. Consequently, visual expressions of illness experiences may be more pertinent to melanoma than other forms of cancer.

Based on the premise that beliefs and emotions about cancer underpin the visual images posted on Instagram, this study used the commonsense model of illness representation [13-15] as an overarching framework to analyze the content of visual cancer communication on Instagram. The commonsense model of illness representation assumes that people are active problem solvers testing lay hypotheses about illness. According to the model, people respond to symptoms and signs of illness by forming cognitive and emotional representations of the illness, which then guide the lay process of coping, planning, and action. Of note, the model posits that illness representations are individualized and not necessarily fact-based; nevertheless, individuals’ illness representations are key determinants of attitude toward risk and managing disease. Primary beliefs about illnesses, including cancer, comprise beliefs about causes, consequences, and control of the illness [13-15].

Along with beliefs, emotions are an important part of illness experiences [15-17]. Martin and colleagues [15] argue that in addition to cognitive responses to and representations of symptoms, people rely on emotional responses to symptoms that direct their coping behavior. Similarly, Gross [16] asserts that emotion expressions are central to cancer experiences ranging from onset to progression. As a result, recent research involving the commonsense model has examined both cognitive and emotional representations as predictors of coping appraisal and illness outcomes [18].

Under this overarching framework, this study used the cognitive appraisal theory of emotions [19,20] to analyze discrete emotional representations in Instagram posts. The cognitive appraisal theory of emotions argues that emotions are outcomes of appraisals of environmental and situational circumstances. Of these circumstances, the experience of cancer can be associated with the negative emotions of fear [21], anger [22], and sadness [23]. Recent cancer survivorship research has discussed the importance of positive emotions such as hope, humor, and happiness. Hope can be an especially important positive emotion in the survivorship process [24]. Snyder and colleagues [25] defined hope as “a positive motivational state that is based on an interactively derived sense of successful (a) agency (goal-directed energy) and (b) pathways (planning to meet goals)” (pg 287). Humor can provide respite for cancer survivors and their loved ones [26]. Finally, the journey of cancer may not be without moments of happiness or joy [27]. We will examine the expressions of these positive and negative emotions.

By investigating Instagram posts about cancer beliefs and emotions, we seek to add new insight to extant research on the commonsense model of illness. Prior research on illness perceptions has used responses prompted by survey or interview questions [28]. On Instagram, people choose to self-express their cancer experiences using images, texts, and other affordances including hashtags. Moving from verbal responses to surveys or interviews, the visual and voluntary expressions of cancer experiences on social media may offer a valuable window for understanding lay sense-making and management of cancer. Self-expression in all settings may be goal directed [29,30], but social media may offer novel opportunities to express what Rogers [31] called “true self.” Rogers’ notion of true self differs from future self or actual self. True self is a present version of self, thus differing from future self, and it is not expressed in social life, thus differing from actual self. Bargh and colleagues [30] asserted that one has the strongest need to express the true self, which is “identity-important and phenomenally real aspects of self,” and that the internet may provide a setting to activate and express it (pg 34).

In addition, we will investigate the visual images particular to the communication of melanoma. These would include images of melanoma or its treatment. Patients’ visual documentation of melanoma or its treatment may hold special meaning to them. Furthermore, these images may increase other users’ engagement in cancer communication on social media in forms such as comments. Our first set of research questions concerned self-expressed beliefs and emotions about melanoma on Instagram:

RQ1: What beliefs are expressed in #melanomasucks posts on Instagram?

RQ2: What emotions are expressed in #melanomasucks posts on Instagram?

RQ3: Are images of melanoma or its treatment included in #melanomasucks posts on Instagram?

### Participative Social Engagement in Self-Expressions of Illness Experience

Self-expression of illness beliefs and emotions on social media invites participative social engagement. It is important to investigate the dynamics between Instagram posts about cancer and the extent and types of network engagement that they generate. This line of research can help shift gears from the current emphasis on the content of social media communication to an expanded research focus that can illuminate the relationship between content characteristics and the participative engagement that they generate.

To investigate the effects of visual expressions of cancer, we focused on participative engagement, as indicated by the number of likes and comments and the types of social support provided in the comments. As an affective reaction, likes may be the most basic form of participative engagement in social media interactions [32]. Comments, which are more cognitively oriented, may indicate a deeper level of engagement because they require more user effort than clicking a like button. Generally, the number of likes and comments are considered indicators of the range of influence of one’s social media post [33]. There is sparse published research investigating the content characteristics predicting likes. Burgeoning research has examined the predictors of comments, focusing on the number of comments from readers of Web-based news [33,34]. In this study, we focus on likes and comments responding to user-generated content containing #melanomasucks.

In addition to likes and comments, the provision of social support may be another form of participative engagement on Instagram. Individuals with chronic illnesses increasingly use social media to connect with others and to seek and provide support [35,36]. Although a growing body of research has examined online social support, a recent review indicates that it has focused on verbal and written exchanges [36]. Research is needed to investigate social support exchanges using affordances on social media platforms. Different Web-based platforms can facilitate or inhibit the seeking and provision of types of support [37]. Users of #melanomasucks on Instagram may constitute what Granovetter [38] termed “weak ties,” individuals with whom close interactions are limited. Weak ties are vital to the cohesion and organizing of communities [38].

Extant literature suggests there are four primary types of social support. They are as follows: esteem, emotional, informational, and instrumental [39,40]. Esteem support refers to the validation of the support receiver’s states and beliefs. Emotional support refers to empathy or encouragement for the receiver’s situation. Informational support refers to advices using facts, data, and references. Instrumental support is provided with tangible assistance. Although growing attention has been paid to Web-based communication of support, little research has examined the connection between the beliefs and emotions expressed by support seekers and the types of support provided. Similarly, sparse research has investigated the relationship between beliefs and emotions and the volume of social engagement they generate, as indicated by the number of likes and comments. We posit that beliefs and emotions represented in Instagram posts will differentially predict likes, comments, and social support, and propose our second set of research questions to explore the effects of content characteristics on these differential types of participative social engagement:

RQ4: What beliefs and emotions predict the number of likes?

RQ5: What beliefs and emotions predict the number of comments?

RQ6: Do images of melanoma or its treatment increase the number of comments?

RQ7: What beliefs and emotions predict the type of social support?

## Methods

### Sampling

The universe from which we draw the sample for this study was #melanomasucks. As discussed above, people use hashtags to label their posts and to ensure the visibility, searchability, and documentability of their posts [4]. We randomly selected 441 publicly available Instagram posts containing #melanomasucks for analysis in this study. At the time of this sampling, there were over 3430 posts using #melanomasucks on Instagram. For random sampling of the posts we used Netlytic [41], which adheres to the privacy policy of Instagram and uses public application program interfaces (APIs) to collect publicly available Instagram posts.

Consistent with the sampling protocol for social media content analyses offered by Kim, Huang, and Emery [42], we assessed the quality of the sample. Quality of social media sampling is assessed with recall and precision [42]. Our use of a hashtag as a sampling frame arrests the issue of recall, which is the degree to which relevant data are retrieved. Precision is the degree to which the retrieved data are relevant. We found that #melanomasucks yielded 95% precision in our sample. The rest of the content analysis procedure was guided by the protocol provided by Neuendorf [43]. Our unit of analysis was each post.

### Measurement

Two coders were extensively trained and they worked together to establish intercoder reliability. We used 10% of the sample to assess the intercoder reliability of each variable. Krippendorff alpha [44] ranged from 0.79 to 1.00. The outcome measures of the number of likes and comments were obtained through API of Instagram. For social support, we assessed the presence or absence of each of the following types of social support: esteem, emotional, information, and instrumental. Consistent with Cutrona and Suhr’s Social Support Behavior Codes [40], these support types were operationalized in the following ways: esteem support compliments, validates, or agrees with the receiver’s perspective expressed in a post; emotional support conveys sympathy, empathy, or encouragement for the receiver’s state; informational support offers facts, data, or references to solve a problem; and instrumental support offers tangible help such as provision of time or donation.

For cancer beliefs, we assessed the presence (1) and absence (0) of beliefs about causes, consequences, and control of melanoma. Under causes, we analyzed the presence or absence of beliefs related to hereditary factors, self behavior, system or institution, natural environment, social environment, built environment, or chance. Under outcomes, we examined the presence or absence of expressions of negative physical, cognitive, emotional, and relational consequences of having melanoma; we also coded for the presence or absence of expressions of positive aspects of having melanoma. Under control, we investigated beliefs related to self and collective actions pertaining to the prevention and control of melanoma. Individuals’ actions included primary prevention (eg, sunscreen), secondary prevention (screening), and treatment; societal actions included raising awareness, fundraising, more research, greater funding allocation, and policy change.

For cancer emotions, we assessed the presence or absence of three negative emotions of fear, anger, and sadness and three positive emotions of hope, joy, and humor. Fear was a depiction of danger or threat; anger was a depiction of wrongful offense; and sadness was a representation of irrevocable loss. Hope was operationalized as a positive emotional state involving goals or planning to meet goals (eg, to get better, to beat cancer, to find a new treatment); humor was a depiction of an effort make fun of or ease a difficult situation; and joy was a variant of happiness that indicated progress toward a goal.

## Results

### Data Analysis Strategy

For research questions 1 and 2, which concerned the characteristics of the content, we computed the frequency and percent distribution of the variables. For research questions 3 and 4, which concerned melanoma beliefs and emotions influencing the number of likes and comments, we used negative binomial regressions. In each model, the number of likes or comments was regressed onto cancer beliefs and emotions. The log number of followers were adjusted as an offset in the regression model. To examine research question 5, predictors of the types of social support, we used a multivariate logistic regression model for each support type. We did not employ multinomial regression to reflect that the social support types were not mutually exclusive. Instead, we used separate binary logistic regression models for each of the four types of social support. In addition to these separate logistic regression models, we used a joint model using group least absolute shrinkage and selection operator (LASSO) logistic regression. To select variables that predict all types of social support, we penalized the total log-likelihood of four logistic regressions for all of the types of support by a group LASSO penalty that encourages the coefficients of a predictor to be zero for all types of support. This helped us select the same set of predictors for all types of support. To determine the penalty-tuning parameter, we selected the group LASSO models with the smallest Bayes information criterion. The number of followers was adjusted in both the separate and group LASSO models.

### Content Characteristics

Research question 1 asked what cancer illness beliefs are expressed in #melanomasucks. Beliefs about controlling melanoma were most frequently expressed (271/441, 61.5%), followed by beliefs about outcomes of melanoma (240/441, 54.4%), which in turn were followed by beliefs about the causes of melanoma (90/441, 20.4%). Table 1 presents the distribution of belief expressions.

Of beliefs about controlling melanoma, awareness raising was most frequently expressed (219/271, 80.8%), followed by treatment (143/271, 52.8%), primary prevention (eg, sunscreen use; 114/271, 42.1%), secondary prevention (eg, screening; 80/271, 29.5%), and fundraising (43/271, 15.9%). Of beliefs about outcomes of cancer, physical consequences were the most frequently expressed (212/240, 88.3%), followed by emotional outcomes (100/240, 41.7%), relational outcomes (56/240, 23.3%), and equal representations of financial consequences (20/240, 8.3%) and positive outcomes (20/240, 8.3%; eg, strengthening of faith during a time of hardship). Of beliefs about causes of melanoma, self behavior was the most frequently expressed (72/90, 80.0%), followed by natural environment (eg, sun exposure, 47/90; 52.2%). A social environment that supports tanning behavior was expressed in 7.8% (7/90) of the posts.

Research question 2 asked what emotions are expressed using #melanomasucks. Hope was the most frequently expressed emotion, such as by providing insight about or expressing trust in a treatment procedure (80/441, 18.1%), followed by fear, such as worry about the recurrence of melanoma (49/441, 11.1%). These were followed by equal representations of humor, such as making light of treatment results, (46/441, 10.4%); sadness, such as remembrance of people who have died from melanoma (46/441, 10.4%); and joy, such as patients expressing enthusiasm and gratitude during treatment (33/441, 7.5%). Anger was a rarely expressed emotion (5/441, 1.1%). Overall, there was a greater number of positive than negative emotional expressions 36.1% (159/441) vs 22.7% (100/441). Table 2 presents the distribution of emotions.

Research question 3 asked whether images of melanoma or its treatment are included in #melanomasucks posts. In total, 21.8% (96/441) of the posts included images of melanoma or its treatment on the face or body parts. Posts showing images of melanoma on the face or body parts were about 4.3% (19/441) of the total posts. Post showing images of melanoma treatment on the face or body parts were about 17.5% of the total posts (77/441).

**Table 1:** Cancer beliefs expressed using #melanomasucks on Instagram (N=441).

Belief			n (%)
**Cause **			90 (20.4)
	Hereditary	0 (0)	
	Self behavior	72 (80.0)	
	System or institution	1 (1.1)	
	Natural environment	47 (52.2)	
	Social environment	7 (7.8)	
	Built environment	0 (0)	
	Luck	2 (2.2)	
**Outcome**			240 (54.4)
	Physical	212 (88.3)	
	Cognitive	0 (0)	
	Emotional	100 (41.7)	
	Relationship	56 (23.3)	
	Financial	20 (8.3)	
	Other negative	1 (0.4)	
	Positive outcomes	20 (8.3)	
**Control**			271 (61.5)
	Prevention	114 (42.1)	
	Screening	80 (29.5)	
	Treatment	143 (52.8)	
	Awareness	219 (80.8)	
	Fundraising	43 (15.9)	
	Research	6 (2.2)	
	Funding allocation	1 (0.4)	
	Guideline change	1 (0.4)	

**Table 2:** Cancer emotions expressed using #melanomasucks on Instagram (N=441).

Emotions		n (%)	
**Positive emotions**			
	Hope		80 (18.1)
	Humor		46 (10.4)
	Joy		33 (7.5)
**Negative emotions**			
	Anger		5 (1.1)
	Fear		49 (11.1)
	Sadness		46 (10.4)

**Table 3:** Effects of belief expressions on the number of likes.

Belief		Estimate (SE)
**Cause**		
	Hereditary	N/A^a^
	Self behavior	−0.67^b^ (0.16)
	System or institution	−2.56 (1.45)
	Natural environment	0.19 (0.18)
	Social environment	−0.12 (0.43)
	Built environment	N/A
	Luck	−1.44 (0.75)
	Other	−0.44 (1.08)
**Outcome**		
	Physical	−0.24^c^ (0.1)
	Cognitive	N/A
	Emotional	0.12 (0.15)
	Relationship	−0.31 (0.18)
	Financial	−1.86^b^ (0.27)
	Other negative	−2.53^c^ (1.04)
	Positive outcomes	0.83^b^ (0.24)
**Control**		
	Prevention	−0.16 (0.12)
	Screening	−0.08 (0.13)
	Treatment	−0.26^c^ (0.11)
	Awareness	0.11 (0.10)
	Fundraising	−0.48^d^ (0.18)
	Research	−0.42 (0.45)
	Funding allocation	−2.89^d^ (1.09)
	Guideline change	−1.36 (1.06)
	Other	−0.65 (0.62)

^a^N/A: not applicable.

^b^*P*<.001.

^c^*P*<.05.

^d^*P*<.01.

### Effects of Content Characteristics

#### Predictors of Likes

Research question 4 asked what beliefs and emotions expressed in #melanomasucks impacted the number of likes. The effects of beliefs on likes are presented in Table 3. Expressions of self behavior as a cause of melanoma were negatively associated with the number of likes (*P*<.001). Expressions about physical (*P*=.03), financial (*P*<.001), and other negative consequences (*P*=.02) significantly decreased the number of likes. In contrast, expressions of positive outcomes of having melanoma significantly increased the number of likes (*P*<.001). Of control beliefs, posts about treatment (*P*=.02), fundraising (*P*=.008), and more research funding allocation (*P*=.008) decreased the number of likes. The effects of emotions on likes are presented in Table 4. Expressions of anger increased the number of likes (*P*<.001), whereas expressions of joy (*P=*.006) and fear (*P*<.001) decreased the number of likes.

**Table 4:** Effects of emotion expressions on the number of likes.

Emotions		Estimate (SE)
**Positive emotions**		
	Hope	0.19 (0.13)
	Humor	0.10 (0.15)
	Joy	−0.02^a^ (0.01)
**Negative emotions**		
	Anger	1.01^b^ (0.26)
	Fear	−0.96^b^ (0.15)
	Sadness	−0.17 (0.15)

^a^*P*<.01.

^b^*P*<.001.

#### Predictors of Comments

Research question 5 asked what beliefs and emotions expressed in #melanomasucks impacted the number of comments. As shown in Table 5, none of the cause beliefs were significantly associated with the number of comments. Of beliefs about outcomes, those about physical consequences of having melanoma significantly increased the number of comments (*P*<.001), whereas beliefs about financial consequences of melanoma decreased the number of comments (*P*<.001). Of control beliefs, those about treatment increased the number of comments (*P*=.03), whereas those about fundraising (*P*=.02) decreased the number of comments. None of the expressions of emotions were associated with the number of comments. Research question 6 asked whether images of melanoma or its treatment increased the number of comments. A significant positive association between such images and the number of comments was found (estimate=0.59, SE=0.18, *P*=.001). Inclusion of melanoma images in posts increased the number of comments.

#### Predictors of Social Support

Research question 7 asked what beliefs and emotions predicted what type of social support. To address this question, we first investigated whether beliefs and emotions predicted the presence or absence of social support using the entire dataset. Next, using only the cases that received social support, we examined predictors of specific kinds of social support.

#### Provision of Support

Of cause beliefs, that self behavior is responsible for melanoma significantly decreased social support comments (estimate=−0.62, SE=0.31, *P*=.048). Of outcome beliefs, those about physical consequences of having melanoma significantly increased social support comments (estimate=0.74, SE=0.22, *P*=.001) and those about relational consequences showed a marginally significant positive effect on social support comments (estimate=−0.70, SE=0.37, *P*=.06). Of control beliefs, those about treatment increased social support comments (estimate=0.58, SE=0.23, *P*=.012). None of the emotions were significantly associated with social support comments.

#### Types of Support

None of the cause beliefs were significantly associated with the types of social support per LASSO. This was confirmed in separate regression models in which no significant association was found in any of the four models for the types of support. Of outcome beliefs, those about physical and emotional consequences were significant per LASSO and confirmed in separate regression models. Beliefs about physical consequences decreased esteem support (estimate=−0.86, SE=0.29, *P*=.003) but increased emotional support (estimate=1.36, SE=0.28, *P*<.001); similarly, emotional consequences decreased esteem support (estimate=−0.82, SE=0.39, *P*=.04) but increased emotional support (estimate=1.34, SE=0.49, *P*=.006). Of control beliefs, those about treatment and fundraising were significant predictors per LASSO, which were confirmed in separate regression models. Expressions about treatment decreased esteem support (estimate=−0.77, SE=0.28, *P*=.005) but increased emotional support (estimate=1.43, SE=0.30, *P*<.001); beliefs about fundraising increased esteem support (estimate=2.29, SE=1.04, *P*=.03) but showed a marginally significant negative association with emotional support (estimate=−0.98, SE=0.51, *P*=.054). Of emotions, fear and sadness were selected per LASSO. Fear decreased esteem support (estimate=−1.35, SE=0.41, *P*=.001) but increased emotional support (estimate=1.56, SE=0.52, *P*=.003); similarly, sadness decreased esteem support (estimate=−0.98, SE=0.39, *P*=.013) but increased emotional support (estimate=1.49, SE=0.51, *P*=.003). No significant associations between the predictors of beliefs and emotions and the informational and instrumental social support types were found.

**Table 5:** Effects of belief expressions on the number of comments.

Belief		Estimate (SE)
**Cause**		
	Hereditary	N/A^a^
	Self behavior	-0.25 (0.24)
	System or institution	−34.4 (1.70e+07)
	Natural environment	0.18 (0.27)
	Social environment	0.63 (0.61)
	Built environment	N/A
	Luck	−0.034 (1.03)
	Other	−1.04 (1.71)
**Outcome**		
	Physical	0.58^b^ (0.15)
	Cognitive	N/A
	Emotional	−0.33 (0.22)
	Relationship	−0.29 (0.27)
	Financial	−2.38^b^ (0.41)
	Other negative	−2.34 (1.67)
	Positive outcomes	0.44 (0.35)
**Control**		
	Prevention	−0.14 (0.17)
	Screening	0.11 (0.19)
	Treatment	0.33^c^ (0.15)
	Awareness	−0.28 (0.15)
	Fundraising	−0.65^c^ (0.27)
	Research	−0.15 (0.69)
	Funding allocation	−2.91 (1.7)
	Guideline change	0.66 (1.38)
	Other	−1.84 (1.14)

^a^N/A: not applicable.

^b^*P*<.001.

^c^*P*<.05.

## Discussion

### Principal Findings

Although ample research has described the content characteristics of text-based cancer communication on social media platforms such as Twitter, few studies have investigated visual communication of cancer and even fewer studies have examined the effects of visual cancer communication on participative engagement outcomes. This study sought to address this gap by investigating the content and effects of #melanomasucks on Instagram. The results of this study provide an important first look at visual communication on cancer on social media. The findings show the self-expressive and social engagement functions that Instagram serves for #melanomasucks users and the areas in which the self-expressive and social engagement functions of the social media platform converge and diverge for users.

The findings of this study may reveal a paradigm shift in cancer communication. Traditionally, magazine editors or television producers have decided what cancer images the public can see in the mainstream media [45]. In the new social media landscape, the lay public decides what to express and share about their cancer experiences. This study found that about 22% of total posts included images of melanoma or its treatment with about 4% showing images of melanoma and 18% showing images of melanoma treatment. These images are rarely found in extant cancer communication in mainstream media. Given that this study found that the inclusion of images increased comments, future research should continue to examine user-generated images of cancer on social media and their effects on public engagement and perceptions.

### Cancer Belief Expressions and Participative Engagement

The results show the aspects of the cancer experience that are meaningful for users of #melanomasucks to express. Users were most interested in expressing the control of melanoma, followed by the outcomes of having melanoma. Causes of melanoma, notably, were least frequently expressed in posts. Of the control beliefs, awareness was the most frequently expressed, followed by treatment, primary and secondary prevention, and fundraising. Of the outcome beliefs, physical consequences were the most frequently expressed, followed by emotional, relational, and financial consequences of having melanoma. Notably, some users expressed positive aspects of cancer experiences (eg, strengthening of faith). Of the cause beliefs, self behavior was the most frequently expressed, followed by natural and social environments.

Convergence between self-expressed and socially engaged beliefs about melanoma was observed where frequent posts increased engagement from other users, for example, posts about melanoma treatment were frequent and they significantly increased comments and emotional social support. Likewise, expressions of the physical consequences of having melanoma were frequent and they increased comments and emotional social support. In addition, expressions of relational consequences of having melanoma were marginally linked to increased emotional social support.

Divergence between self-expressive and social engagement functions of #melanomasucks was observed where frequent posts did not foster engagement from other users; for example, whereas self behavior as a cause of melanoma comprised 80% of cause-related beliefs, expressions of this belief decreased the number of likes and social support comments. Similarly, none of the cause beliefs were significantly associated with the number of comments. In addition, posts about financial consequences of having melanoma decreased the number of likes and comments. Although posts about personal experiences (eg, treatment and physical consequences) engaged users with increased comments and emotional social support, posts that were less personal (eg, awareness, fundraising) decreased the number of comments.

### Cancer Emotion Expressions and Participative Engagement

Intriguing patterns of results were obtained for the research questions concerning emotions. Regarding self-expression of emotions, users of #melanomasucks were more interested in expressing positive emotions (ie, hope, humor, and joy) than negative emotions (ie, anger, fear, and sadness) and of the six emotions, hope was the most frequently expressed. These findings point to the need for more research on the roles of positive emotions and positive emotional expressions in cancer management.

A notable contrast between positive and negative emotions emerged in relation to the self-expressive and social engagement functions. Positive emotions appear to serve more self-expressive functions than social engagement functions because they did not increase likes, comments, or social support. Although hope was the most frequently expressed emotion, it did not increase any of the indicators of engagement including likes, comments, or social support. Likewise, humor, which was apparently expressed in users’ efforts to make light of difficult melanoma-related situations, was not associated with the number of likes or comments. Moreover, expressions of joy decreased the number of likes. Somewhat similarly, posts that described positive aspects of cancer experiences increased the number of likes but not comments or social support. In comparison, although negative emotions were less frequently expressed, they enjoyed more social engagement from other users than positive emotions; for example, anger, which was the least frequently expressed emotion, significantly increased the number of likes. Although fear decreased the number of likes, it received emotional social support; expressions of sadness were unassociated with likes but they increased emotional social support.

### Implications for Theory and Research

The results provide important implications for theory and research on the commonsense model of illness and participative engagement on social media. Extant research on illness perceptions using the commonsense model has employed a validated and standardized protocol [28] though interviews or surveys. Illness perceptions gathered through visual self-expressions on Instagram offer new insight on lay sense-making of cancer experiences. The control and outcomes of cancer were more important for users of #melanomasucks to express compared with the causes of cancer. Although considerably less research has examined positive emotions associated with cancer management than negative emotions, the findings show that it was positive emotions that more #melanomasucks users wanted to express. Future cancer communication research should allocate greater attention to investigating the meaning and impact of positive emotions in cancer experience and management.

The convergence and divergence of the self-expression and social engagement functions of #melanomasucks found in this study may point to an important new direction for future research on participative engagement on social media. The pattern of convergence and divergence that emerged appears to depart from the normative expectations in face-to-face interactions; for example, posts about financial consequences of having melanoma decreased the number of likes and comments. Similarly, expressions of positive emotions received little social engagement. These results add to the growing body of research examining the differences between online and offline communication behavior [46].

Also noteworthy is the divergence between self-expressions of positive emotions and social support received. Arguing that emotions are socially interdependent by nature and elicit social sharing, Rime’ [47] posited that negative emotions stimulate cognitive work, social interaction, and conversation, and that positive emotions can enhance subjective well-being when savored and shared. The findings of this study appear to resonate with the theorizing of Rime’, contributing to the understanding of the social engagement functions of emotions and where and how the effects of negative and positive emotions may deviate. In addition, the findings showed that the predictors of likes and comments differed from those of social support. More research should examine the predictors of these differential forms of social engagement and the effects of this engagement on the recipients and providers.

### Limitations and Suggestions for Future Research

As the first investigation of visual cancer communication on social media, this study was limited to one cancer, melanoma, and one hashtag, #melaonomasucks. Building on this study, future research should examine whether the patterns of content and effects identified in this study are similar for other cancers and other cancer hashtags, where the similarities and differences arise in the expression of causes, outcomes, control, and emotions, and the participative engagement that these expressions may elicit. Using the framework developed in this study, future studies could employ a larger sample.

### Conclusion

This study provides an important first look at the content and effects of visual cancer communication on social media and a conceptual basis that future investigation of visual communication of other cancers can utilize. The convergence and divergence identified between self-expressions of cancer beliefs and emotions and the social participative engagement they foster offer new insight and directions for extending the research on cancer illness perceptions and for developing conceptual frameworks for explaining and predicting participative engagement in cancer communication on social media.

## Abbreviations

API: application program interface
LASSO: least absolute shrinkage and selection operator
